# Clinical utility of methionine restriction in adenosine kinase deficiency

**DOI:** 10.1002/jmd2.12238

**Published:** 2021-07-27

**Authors:** Najmah Almuhsen, Simon‐pierre Guay, Marie Lefrancois, Cheryl Gauvin, AL Qasim Al Bahlani, Najma Ahmed, Christine Saint‐Martin, Tommy Gagnon, Paula Waters, Nancy Braverman, D. Buhas

**Affiliations:** ^1^ Division of Genetics, Department of Specialized Medicine McGill University Health Center Montréal Quebec Canada; ^2^ Division of Pediatric Gastroenterology, Department of Pediatrics McGill University Health Center Montréal Quebec Canada; ^3^ Department of Radiology McGill University Health Center, Montreal Children's Hospital Montreal Quebec Canada; ^4^ Medical Genetics Service, Department of Pediatrics and of Laboratory Medicine Université de Sherbrooke‐CHUS Sherbrooke Quebec Canada; ^5^ Department of Human Genetics McGill University Montréal Quebec Canada; ^6^ Department of Pediatrics McGill University Montréal Quebec Canada

**Keywords:** ADK deficiency, adenosine, gestational alloimmune disease, hypermethioninemia, liver dysfunction in metabolic disease, methionine, purines

## Abstract

Adenosine kinase (ADK) deficiency is a rare autosomal recessive inborn error of metabolism involving the methionine and purine metabolic pathways. Prior reports show that most patients present in infancy with jaundice, hypotonia, developmental delay, and mild dysmorphic features. Characteristic biochemical findings included hypoglycemic hyperinsulinism, cholestasis, elevated liver functions, methionine, S‐adenosylhomocysteine, and S‐adenosylmethionine, with normal or mildly elevated homocysteine level. Brain imaging demonstrated atrophy, hydrocephalus, and delayed myelination. There are 26 reported patients of ADK deficiency, of which 14 patients were placed on a methionine‐restricted diet. Clinical improvement with methionine restriction was not well described.

**Case report:**

We report an infant who presented at birth with persistently elevated ammonia (100‐163 μmol/L), hypoglycemia, cholestasis, and liver dysfunction. The initial metabolic and genetic work‐up was nondiagnostic, with only a mildly increased plasma methionine level (51 [<38 μmol/L]). Iron depositions in the liver and in lip mucosa led to suspicion of gestational alloimmune liver disease. Immunoglobulin therapy and exchange transfusion treatments demonstrated transient clinical and biochemical improvements. However, subsequent episodes of acute liver failure with development of neurological abnormalities led to further evaluation. Metabolic studies showed a 25‐fold increase in plasma methionine level at 8 months of life (1022 [<38 μmol/L]) with white matter abnormalities on brain MRI. Expanded molecular testing identified the disease. Urinary purines profile showed elevations of adenosine and related metabolites. Introduction of a low‐methionine diet resulted in rapid clinical amelioration, improvement of brain MRI findings, and normalization of liver functions and methionine levels.

**Synopsis**

ADK deficiency should be in the differential of neonatal and recurrent liver dysfunction, even when plasma methionine levels are not significantly increased. A low‐methionine diet should be trialed to determine clinical impact in patients with ADK deficiency.

## 1 INTRODUCTION

Adenosine kinase (ADK) deficiency (OMIM #614300) is an autosomal recessive inborn error of methionine and adenosine metabolism. A total of 26 cases have been reported in the literature so far.^1^, ^2^, ^3^, ^4^, ^5^ This panethnic condition has been described in Swedish, German, Malaysian, Turkish, and Saudi patients.^1^, ^5^ ADK deficiency is caused by pathogenic biallelic variants in the *ADK* gene which encodes the ADK enzyme involved in the phosphorylation of adenosine to adenosine monophosphate (AMP).^6^ ADK is also involved in methionine metabolism by removing adenosine, which is critical for the proper function of S‐adenosylhomocysteine (SAH) hydrolase and transformation of SAH into homocysteine. Accumulation of adenosine can lead to inhibition of SAH hydrolase with subsequent accumulation of SAH, S‐adenosylmethionine (SAM), and methionine.^6^

Hypermethioninemia is a biochemical marker of inherited inborn errors of metabolism involving the methylation cycle such as MATI/III deficiency, glycine N‐methyltransferase deficiency, SAH hydrolase deficiency, and homocystinuria due to CBS deficiency.^2^, ^7^ It is also associated with several genetic‐metabolic disorders in which the mechanism of methionine elevation is less direct (eg, mitochondrial disorders, citrin deficiency or fumarylacetoacetate hydrolase deficiency/tyrosinemia type 1).^7^ Hypermethioninemia can also be secondary to liver disease or prematurity especially in the neonatal period.^7^

The patients so far described with ADK deficiency presented early in the neonatal period with sepsis‐like symptoms, respiratory difficulties, jaundice and dysmorphic features, especially frontal bossing and hypertelorism.^3^ All reported patients also had neurologic manifestations such as developmental delay, hypotonia, and seizures.^1^ In addition, cardiac anomalies have been reported with increased frequency such as, but not limited to, atrial septal defect (ASD), ventricular septal defect (VSD), and patent ductus arteriosus (PDA).^3^, ^6^ Characteristic biochemical findings included hypermethioninemia with elevated SAH and SAM but normal or mildly elevated homocysteine level.^1^, ^6^, ^8^ Other common biochemical findings include hyperinsulinemic hypoglycemia and recurrent hepatic insult with high liver enzymes and cholestasis secondary to infections.^4^, ^9^ Neuroradiological imaging often showed CNS involvement with brain atrophy, hydrocephalus, and delayed myelination.^1^, ^4^ Myelination ultimately improved with later onset of nonspecific white matter changes.^3^, ^8^

The diagnosis of ADK deficiency is suspected based on biochemical findings and can be confirmed by molecular analysis showing pathogenic bi‐allelic variants in the *ADK* gene.^3^ Management consists of supportive treatments with a multidisciplinary team and periodic monitoring of neurological and hepatic functions.^2^ No curative treatment is reported for ADK.^3^ However, our experience with this patient and our literature review suggest that a low‐methionine diet may have a major impact on the clinical evolution in at least some patients with ADK deficiency.

## 2 CASE REPORT

The patient is currently a 2‐year‐old boy who is the first child of nonconsanguineous Filipino parents. He was born at 37 weeks of gestation by spontaneous vaginal delivery from a 36‐year‐old healthy mother. The pregnancy was complicated by mild gestational diabetes well controlled with diet with normal second trimester ultrasounds. The delivery was uncomplicated, and APGARs were 8 and 9. His birth weight was 2.6 kg (5th percentile), length was 48 cm (15th percentile), and head circumference was 34 cm (30th percentile). Borderline low serum glucose levels in the first 12 hours of life were managed with feeding. Within the first day of life, he required phototherapy for jaundice (total bilirubin of 319, normal <263 μmol/L). On neonatal physical exam, he was noted to be hypotonic, and a cardiac murmur was heard. An echocardiogram showed a small ASD and a large PDA that closed spontaneously soon after birth.

Initial laboratory evaluation showed abnormal coagulation profile (INR 4.8) with hyperammonemia (169) [normal range < 100 μmol/L for newborn], glutamine 500 [473‐692 μmol/L], citrulline 54 [14‐32 μmol/L]), and normal liver enzymes. He was referred to the genetics service for a positive newborn screening test (sample obtained at age 34 hours), suggesting L‐3 hydroxyacyl‐CoA dehydrogenase (LCHAD). This was based on mild elevations of C16‐OH and C18:1‐OH; however, the bloodspot also showed multiple nonspecific elevations of other acylcarnitines and the overall profile was not typical of LCHAD deficiency or any other fatty acid oxidation disorder. Meanwhile, a plasma acylcarnitine profile (age 3 days) was normal except for mild elevations of several nonhydroxylated long chain acylcarnitines. These were considered secondary to immaturity and nutritional status and became nearly normal in repeat specimens within a week. The possibility of a fatty acid oxidation disorder was subsequently excluded by molecular analysis of all relevant genes. Galactosemia was unlikely, given normal galactose‐1‐phosphate uridylyltransferase (GALT) enzyme activity, galactose, and galactose‐1‐phosphate in the newborn bloodspot. A persistent moderate increase in methionine levels (51‐110 μmol/L) was noted in the plasma amino acid profile within the first week of life but was thought to reflect the neonatal liver dysfunction.

Because of persistent hyperammonemia, the patient was started on an ammonium scavenger (sodium benzoate) and ammonia levels normalized. A gene panel for hyperammonemia was nondiagnostic. Within the first week of life, he developed additional signs of liver injury with increased direct bilirubin, abnormal coagulation profile, and elevated liver enzymes. High ferritin (5350 [6‐11 μg/L]) and alpha‐fetoprotein (154  348 [0‐80 000 ng/mL]) levels suggested a diagnosis of gestational alloimmune liver disease (GALD). A lip biopsy showed glandular iron deposition in a single minor salivary gland and abdominal MRI showed normal liver anatomy, with evidence of iron deposition in the liver but no extra‐hepatic iron deposition. In the context of neonatal liver failure and a working diagnosis of GALD, the patient received IVIG on days 5 and 10 of life and an exchange transfusion on day 10 of life. His clinical status and laboratory findings, improved remarkably and he was discharged home at 1 month of age. A cholestasis gene panel was performed and did not identify any abnormality.

**FIGURE 1:**
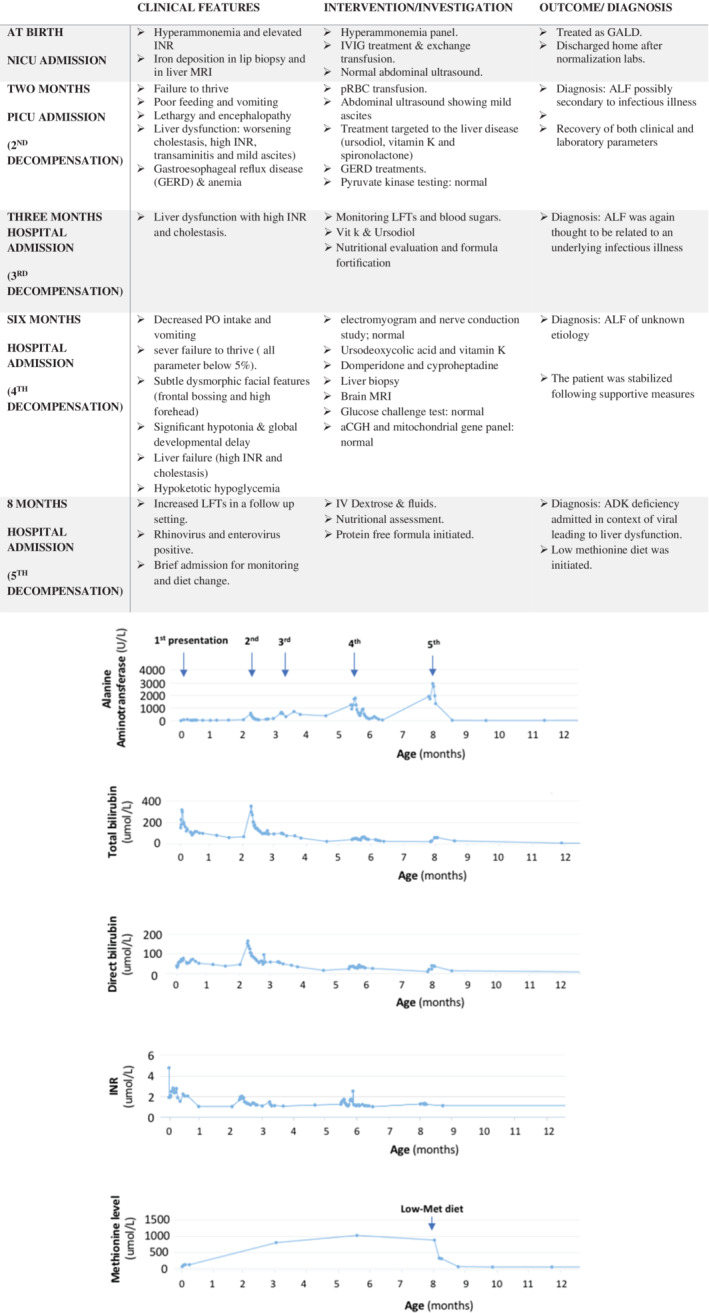
Clinical presentation and investigation. A) Table; hospitalization summary including age at presentation, clinical status and intervention during each admission. ALF, acute liver failure; INR, International normalized ratio; MRI, magnetic resonance imaging; NICU Neonatal Intensive Care Unit; PICU, Pediatric Intensive Care Unit; pRBC, packed red blood cells. B) Evolution of liver profile and methionine levels since the neonatal period; This panel shows the variation in liver enzyme (alanine aminotransferase shown in U/L), bilirubin total/direct (μmol/L), international normalized ratio and plasma methionine level (μmol/L) since the first presentation during the neonatal period. Since birth, he has had five episodes of acute liver injury. After the patient was diagnosed with ADK deficiency and started on a low‐methionine diet (at age 8‐9 months), no episode of acute liver injury was observed, and his liver profile and methionine level normalize

The baby was re‐admitted at the age of 2, 3, and 6 months with a clinical picture related to liver injury of variable severity and was evaluated and treated closely by a multidisciplinary team. On admissions at age 2 and 3 months, it was hypothesized that he had an acute viral illness causing acute liver injury in the context of a previously injured liver from GALD. Figure 1A summarizes symptoms, investigations, and interventions done during those hospitalizations.

On his third re‐admission at 6 months of age, a liver biopsy was done and showed nonspecific findings of cholestatic hepatitis with preserved native bile duct structures and no significant iron deposition. There was evidence of chronic injury with stage 3 fibrosis. These pathological features did not provide an etiology for the patient's clinical presentation. Further metabolic investigations were pursued and revealed a persistent marked hypermethioninemia (1022 μmol/L) (Figure 1B).

**FIGURE 2:**
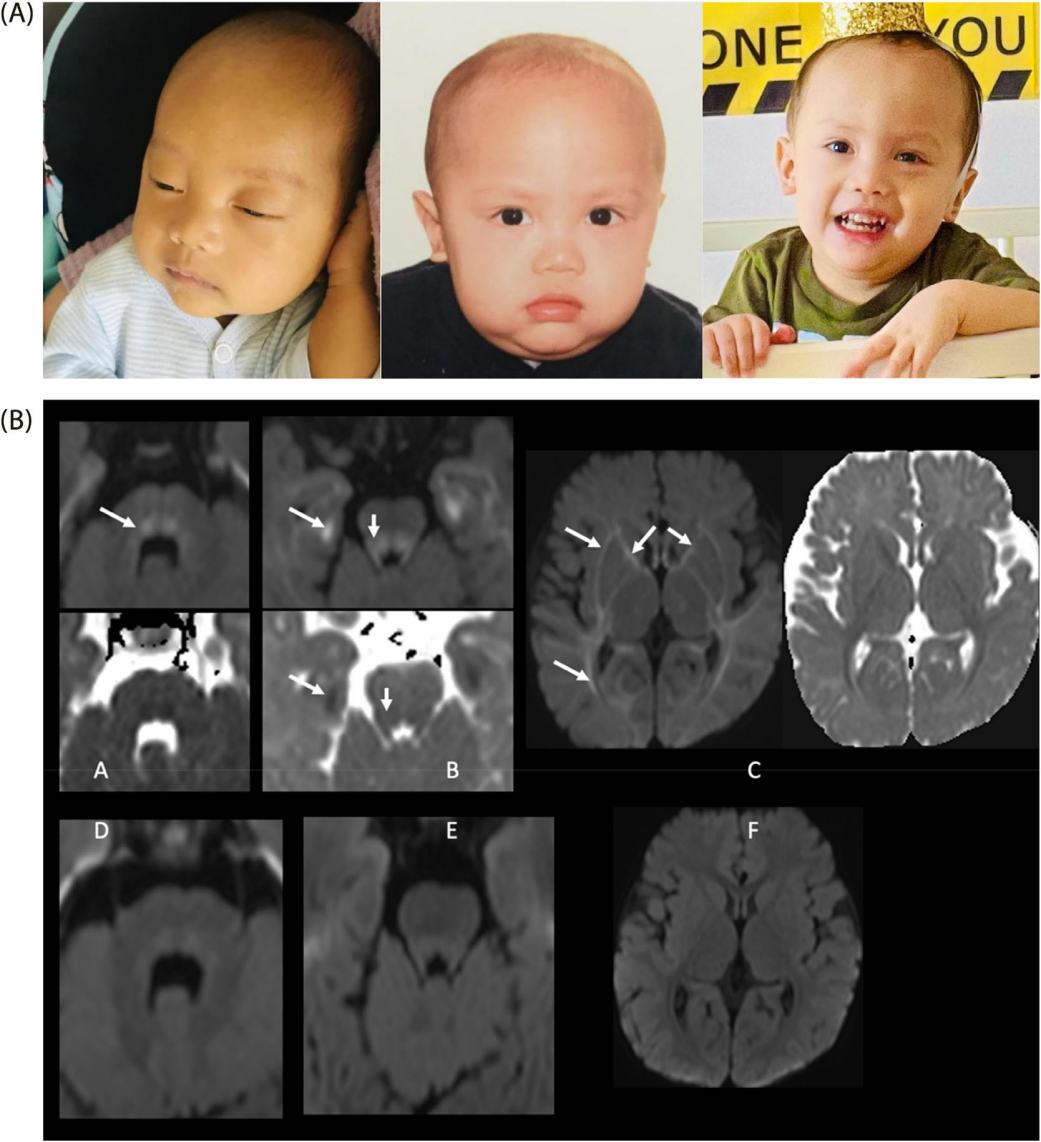
Dysmorphic features and brain MRI findings. A) Picture on the far left shows our patient at 3 months of age with jaundice, frontal bossing, macrocephaly and coarse face. Picture in the middle shows our patient at 11 months of age after initiation of low‐methionine diet. We can see the same dysmorphic features in addition to body‐built improvement following weight gain. Picture on the right is his second birthday, hypotonia can be appreciated in the hands. B), Brain MRI finding showing diffusion imaging (b = 1000) and ADC map at 6 (A, B, C) and 12 (D, E, F) months of age. Panel A showed descending dorsal fibers of the tegmentum with high signal (white arrow) that resolved at 12 months of age (Panel D). Panel B shows restricted diffusion in the mesial temporal subcortical white matter as well as in the tegmentum and superior cerebellar peduncles (white arrows) that resolved at 12 months of age (Panel E). Panel C shows mild restricted diffusion in the supratentorial white matter involving the internal and external capsules, optic radiations and subcortical white matter (white arrows) that resolved at 12 months of age (Panel F)

Brain MRI performed at age 6 months in the context of motor developmental delay, showed extensive abnormal restricted diffusion (involving the supratentorial white matter, hippocampi, optic radiations, thalami, brainstem, superior and middle cerebellar peduncles, and superior cerebellar white matter) which was highly suspicious for a metabolic etiology (Figure 2B). A repeat brain MRI at 13 months of age, while on a methionine‐restricted diet, showed significant interval improvement in the brain maturation with residual mild delayed myelination, milder degree of residual restricted diffusion, and returned to a normal T1 and T2 signal for age with no brain volume loss (Figure 2B).

The diagnosis of ADK deficiency was done through an expanded molecular analysis of more than 10  000 genes, which revealed that the patient was homozygous for a novel frame shift variant in exon 10 of the *ADK* gene; NM_001123.3 (c.896delG; p. Gly299Glu fsX18). This likely pathogenic variant as per ACMG criteria^10^ was not previously reported in patients with ADK deficiency and is not observed in large population cohorts.^11^ It was detected in the heterozygous state in both parents.

Urine purines and pyrimidines profiles also were consistent with this diagnosis, showing persistent substantial increases in adenosine (Supplementary Table 1). There were also moderate increases of two other purine metabolites: inosine and aminoimidazole carboxamine riboside (AICar) (Supplementary Table 1).

Given the diagnosis of ADK deficiency, the patient was started on a low‐methionine diet (15‐20 mg/kg/day) at the age of 8 months, after having a fifth episode of acute liver injury with elevated liver enzymes and coagulopathy. His functional developmental age before initiating the diet corresponded to a 4‐month‐old child. He was also on nasogastric tube feeding due to failure to thrive. The low‐methionine diet consisted of a mix of 40 g pregestimil, 22 g XMet Analog, and 97 g Pro‐Phree formula mixed in 685 mL of water. This recipe was regularly adjusted in view of his weight gain and methionine levels. After a dramatic improvement of all liver functions and methionine levels within 2 weeks of methionine restriction, he was also switched to bottle and puree feeds (Figure 1B).

Immunological evaluation was done at 13 months given the history of recurrent viral illness. T/B cell enumeration, immunoglobulins (IgA, IgE, IgG, IgM) and specific antibody responses to tetanus, diphtheria, Hib, and HBV were adequate. Testing for tuberculosis antigen showed poor response to mitogen, which implied decreased T‐cell function. Lymphocyte proliferation after stimulation to mitogens was subnormal to phytohemagglutinin but normal to concanavalin A and pokeweed mitogen.

Currently, at 2 years of age, he has had no further episodes of acute liver injury since starting the low‐methionine diet. He receives lansoprazole, cyproheptadine, and domperidone for control of gastroesophageal reflux disease and suboptimal appetite. Growth parameters at this visit showed weight of 9.6 kg (3rd percentile), height of 80 cm (first percentile), and relative macrocephaly with head circumference of 49.6 cm (75th percentile). Physical examination was remarkable for subtle dysmorphic facial features (Figure 2A) and improved but persistent generalized hypotonia. Moreover, significant progress in his neuro‐development was achieved. He started to walk with support, was able to count in two languages and use three‐word sentences.

## 3 DISCUSSION

Neonatal acute liver failure (NALF) can be caused by multiple etiologies, of those GALD is a common cause, while individual inborn error of metabolism (IEM) are rarer.^12^ Our patient had a clinical picture mimicking GALD in the neonatal period. In the absence of another diagnosis and a potential treatment for GALD, the baby was treated as such. In retrospect, we did not repeat the abdominal MRI to determine if liver iron deposition improved. In the previously reported cases of ADK deficiency, there were no mention of iron deposition or GALD. It is possible that the hepatic and extrahepatic iron storage observed was secondary to a chronic liver disease, suggesting a prenatal onset of this condition.^12^

Our patient had multiple episodes of acute liver decompensations triggered by infection, suggesting that the underlying pathophysiology of repetitive liver injury was possibly immune related, as sensitivity to infection was previously reported.^4^, ^9^ Adenosine deaminase deficiency (ADA), another condition with adenosine accumulation, is associated with severe combined immunodeficiency as well as cognitive impairment, hepatic dysfunction, and neurodevelopmental deficits,^13^ features that overlap with ADK deficiency. Evaluation of the immune status of our patient showed some abnormalities. Further studies and review of other cases will be required to elucidate impact of this disorder on immune system and liver injury.

There are 26 cases of ADK deficiency described, and all patients had an abnormal brain MRI and neurological outcome (see Supplementary Table S2). The underlying neuropathology is not well understood, and it is possible that increased methionine and adenosine A2A receptor play a role in the brain synaptic plasticity.^8^, ^14^ The influence of elevated plasma methionine levels on MRI findings (ie, immaturity, delayed myelination) was previously reported^8^, ^13^, ^15^, ^16^ and considered a reflection of intracellular brain edema, with a characteristic pattern. Interestingly, the normalization of plasma methionine and decrease in adenosine levels noted in our patient post treatment were associated with the marked improvement of brain imaging and neurodevelopmental status. The pattern and mechanism for the observed improved MRI findings are still uncertain and are not necessarily seen in other diseases within the same pathway. For example, in MAT I/III deficiency even if plasma methionine levels were controlled, the abnormal signal intensity in the brain MRI lesions will persist^17^ suggesting possible adverse effect of adenosine in ADK deficiency. This highlights the potential clinical impact of substrate reduction therapy in prevention of neurological complications in ADK deficiency.

The literature suggests that substrate reduction therapy through a methionine‐restricted diet had variable outcomes in ADK deficiency.^1^ A consensus statement for inherited methylation disorders^2^ recommends considering methionine restriction as a therapeutic option. Review of treatment status, hepatic features and neurological outcomes in previously published patients (Supplementary Table S2), suggests better outcomes with earlier treatment (12/27 showed hepatic improvement and 11/27 neurodevelopmental improvement). However, the methionine‐restricted diet is not associated with complete recovery of the clinical picture, pointing to other possible pathophysiologic factors, such as high adenosine level.

As previously described for several other patients,^3^, ^6^ our patient had increased urinary adenosine. We also observed elevations of two other purine metabolites, that is, inosine and AICAr. These were not previously reported in ADK deficiency, but both can plausibly be considered secondary to the accumulation of adenosine in this condition. Inosine is produced by the action of adenosine deaminase, while AICAr is an upstream metabolite in the de novo purine synthesis pathway. These results suggest that urinary purine analysis might provide a useful screening test for ADK deficiency. However, for some other reported patients,^3^ the observed urinary adenosine concentrations were only slightly elevated or were in the upper normal range. Several factors could influence such observations. Patients with a complete deficiency of ADK reflecting a “null genotype,” as is likely the case for our patient, would probably tend to have greater accumulation of adenosine than patients in whom the genotype permits some residual ADK activity. It is also plausible that the concentration of adenosine in a given specimen could be influenced by the nutritional status at the time of sample collection. In situations where the increase in urinary adenosine is only subtle, this is more likely to be identified as abnormal if a highly sensitive and specific analytical method is used, such as the liquid chromatography‐tandem mass spectrometric assay, than it would be if using a traditional method involving ultra‐violet detection. For all of these reasons, it is important to note that a normal urinary adenosine result does not rule out a diagnosis of ADK deficiency.

## 4 CONCLUSION

Our case report provides an in‐depth investigation of a single case of ADK deficiency due to a novel genetic variant, along with comparison to previously published cases and strengthens the use of a low‐methionine diet as a therapeutic option for this disease. Our patient uniquely mimicked GALD in the context of a clinical presentation of neonatal acute liver failure. We also demonstrate that substrate reduction therapy through a methionine‐restricted diet in this patient was effective in normalizing methionine levels and was associated with rapid recovery of liver function, and potentially contributed to the improved neurodevelopmental outcome. We also show that analysis of urinary purines can contribute to screening and diagnosis of ADK deficiency. As more cases of ADK deficiency are documented, they will contribute to a better understanding of the natural history of this rare inborn error of metabolism and clearly show that ADK deficiency should be consider in the differential diagnosis of hypermethioninemia in the context of liver failure.

## CONFLICT OF INTEREST

All authors declare that they have no conflict of interest.

## INFORMED CONSENT

All procedures followed were in accordance with the ethical standards of the responsible committee on human experimentation (institutional and national) and with the Helsinki Declaration of 1975, as revised in 2000 (5). Informed consent was obtained from the patient's family for being included in the study, including the use of the child's photography. Additional informed consent was obtained from the patient for whom identifying information is included in this article. This article does not contain any studies with human or animal subjects performed by the any of the authors.

## AUTHORS CONTRIBUTION

D. Buhas, Marie Lefrancois, Cheryl Gauvin, Najma Ahmed, Simon‐pierre Guay, Najma Ahmed, and AL Qasim Al Bahlani were involved in the direct clinical care of our reported patient. Nancy Braverman reviewed the presentation and associated high methionine with specific brain imaging findings. Christine Saint Martin reviewed the brain imaging. Tommy Gagnon established and performed the method for urinary purine profile analysis. P. J. Waters provided laboratory data and interpretation of biochemical profiles. Najma Ahmed wrote the case report; all authors read and agreed to the published version.

## Supporting information

**Supplementary Table 1:** Urinary purine and pyrimidine profiles

**Supplementary Table S2:** Summary of previously reported patient with outcomes
