# Supplementary material for: Clinical utility of methionine restriction in adenosine kinase deficiency
Source: JIMD Rep. 2021 Jul 27;61(1):52–9. doi: 10.1002/jmd2.12238 (PMC8411109; doi:10.1002/jmd2.12238)
Supplement: Supplementary file 1 — Supplementary Table 1: Urinary purine and pyrimidine profiles [file JMD2-61-52-s002.docx]

**Supplementary Table 1**. Urinary purine and pyrimidine profiles

|  | At age 12 months | | At age 19 months | | Reference range |
| --- | --- | --- | --- | --- | --- |
|  | Conc. | Fold  elevation | Conc. | Fold  elevation |  |
| Adenosine | 50 | 25X | 33 | 16X | < 2 |
| Inosine | 19 | 5X | 16 | 4X | < 4 |
| AICAr | 20 | 7X | 15 | 5X | < 3 |
| Other purines (12 metabolites) | Essentially normal | NA | Essentially normal | NA |  |
| Pyrimidines  (13 metabolites) | Essentially normal | NA | Essentially normal | NA |  |

A profile of 15 purine metabolites and 13 pyrimidine metabolites was analysed in urine, using a liquid chromatography-tandem mass spectrometry (LC-MS/MS) method developed within the biochemical genetics laboratory of CHUS. The quoted reference ranges are applicable to patients aged 0 – 24 months. Conc., concentration in mmol/mol creatinine. AICAr, aminoimidazolecarboxamide riboside
