# Supplementary material for: Clinical utility of methionine restriction in adenosine kinase deficiency
Source: JIMD Rep. 2021 Jul 27;61(1):52–9. doi: 10.1002/jmd2.12238 (PMC8411109; doi:10.1002/jmd2.12238)
Supplement: Supplementary file 2 — Supplementary Table S2: Summary of previously reported patient with outcomes [file JMD2-61-52-s001.docx]

| Publication | Patient | Methionine level before diet (mmol/L) | Dietary restrictions  mg/kg/day methionine-age diet started | Hepatic dysfunction:  Elevated liver enzyme/ liver biopsy description /improvement with diet | Seizure disorder/ age of onset | Neurodevelopmental delay/ improvement with diet |
| --- | --- | --- | --- | --- | --- | --- |
| Labrune et al, 1990  Biochemical diagnosis  Tunisian Family  3 sisters | 1 | 1000 | 35 mg - 5 months | +/ steatosis, moderate portal fibrosis/+ | Refractory seizures/4 years | + (sever)/- |
|  | 2 | 665 | 30-40 mg -days of life 15 | +/ diffused steatosis, no portal fibrosis/+ | - | + (Moderate)/+ |
|  | 3 | 600 | 50- 12 mg-day of life 6 | +/scattered and mild steatosis/+ | - | + (moderate)/+ |
| Bjursell et al, 2011  (3 families)  1 Swedish, and 2 Malaysian | 4 | 455 | n.a. | +/ steatosis/n.a. | +/1 year | +/n.a. |
|  | 5 | 886 | n.a. | +/ n.a./n.a. | +/1year | +/n.a. |
|  | 6 | 800 | n.a. | +/ cholestasis/n.a. | +/2 years | +/n.a. |
|  | 7 | 550 | n.a. | +/ cholestasis/n.a. | +/10 months | +/n.a. |
|  | 8 | 800 | n.a. | +/ cholestasis/n.a. | +/ 16 months | +/n.a. |
|  | 9 | 600 | n.a. | +/ cholestasis/n.a. | +/2 years | +/n.a. |
| Staufner et al, 2016  7/11 treated with methionine restriction (15-20 mg per kg)  Polyethnic group  (8 families) | 10 | 107 | Not treated | +/ liver fibrosis/- | +/5 months | +/n.a. |
|  | 11 | 400 | n.a.- 5 days during neonatal period | +/ liver fibrosis/- | +/4 months | +/n.a. |
|  | 12 | 867 | n.a.-7 months till 4.9 years | +/ no liver fibrosis/+ | +/ 4.8 years | +/+ |
|  | 13 | 910 | n.a.-First months till 1.2 yrs. | +/ liver fibrosis/+ | +/8.1 years | +/+ |
|  | 14 | 162 | Interrupted due to diarrhea | +/ n.a./- | +/6month | +/n.a. |
|  | 15 | 135 | birth | +/ n.a./+ | - | +/+ |
|  | 16 | 1100 | 2.5 yrs. | +/ liver fibrosis/+ | - | +/+ |
|  | 17 | 350 | 3.25 -4.25 yrs. | +/ n.a./ineffective | - | +/n.a. |
|  | 18 | n.a. | n.a. | -/ n.a./n.a. | +/1year | +/n.a. |
|  | 19 | n.a. | n.a. | -/ n.a./n.a. | +/ 1year | +/n.a. |
|  | 20 | 436 | n.a. | +/ n.a./n.a. | - | +/n.a. |
| Shakiba et al, 2016 (Persian) | 21 | 1200 | 17-20 mg - Since age 1 | +/ stage 4 fibrosis/+ | - | +/+ |
| Alhusani et al. 2019 Saudi | 22 | 1500 | Not treated | +/ fibrosis/ n.a. | - | +/n.a. |
| Kuptanon et al. 2019 Thailand | 23 | 11-42 | Protein restriction at 2 years | n.a. / n.a./ + | +/5 months | +/+ |
| Becker et al, 2020  2 families | 24 | 242 | Untreated | +/ liver cirrhosis/n.a. | - | +/n.a. |
|  | 25 | 143 | 20mg- at 4 months | +/ n.a./+ | - | +/+ |
|  | 26 | 663 | 20mg- at 7 months | +/ n.a./+ | - | +/+ |
| Current case | 27 | 1022 | 15-20 mg/kg/day- 8 months | +/ stage 3 fibrosis/+ | - | +/+ |
| Total | 27 |  | 14 | 26/12 | 12 | 27/11 |

Summary of previously reported patients; methionine levels, status of treatment, liver phenotype, seizure, neurodevelopmental outcome and effects of dietary intervention on the liver and neurological outcome. Of note 14 patients are reported to have been treated with methionine restriction 12 had improved liver related problems and 11 had improved neurodevelopmental outcome, 12 had seizure disorders with only 3 of them belong to the treated group. (n.a.; not available; yrs.: years).
